# The face of Non‐photosensitive trichothiodystrophy phenotypic spectrum: A subsequent study on paediatric population

**DOI:** 10.1002/mgg3.2501

**Published:** 2024-08-09

**Authors:** Giulia Pascolini, Martina Lipari, Federica Gaudioso, Luca Fania, Giovanni Di Zenzo, Biagio Didona

**Affiliations:** ^1^ Genetic Counselling Service Istituto Dermopatico dell'Immacolata, IDI‐IRCCS Rome Italy; ^2^ Rare Skin Diseases Center Istituto Dermopatico dell'Immacolata, IDI‐IRCCS Rome Italy; ^3^ Precision Medicine and Pharmacogenomics Unit Sandro Pertini Hospital Rome Italy; ^4^ Medical Genetics Division Foundation IRCCS Cà Granda Ospedale Maggiore Policlinico Milan Italy; ^5^ Dermatology Clinic Istituto Dermopatico dell'Immacolata, IDI‐IRCCS Rome Italy; ^6^ Molecular and Cell Biology Laboratory Istituto Dermopatico dell'Immacolata, IDI‐IRCCS Rome Italy

**Keywords:** DeepGestalt, GestaltMatcher, Non‐photosensitivity, trichothiodystrophy

## Abstract

**Background:**

Non‐photosensitive trichothiodystrophies (TTDs) are a diverse group of genodermatoses within the subset of conditions known as “sulphur‐deficient brittle hair” syndromes. A part of them has only recently been identified, revealing novel causative genes and very rare phenotypes of these genetic skin disorders. At the same time, the molecular basis of previously published and unresolved cases has been revealed through the introduction of innovative genetic techniques. We have previously described the facial phenotype of patients with the Photosensitive form of TTD during childhood. This study marks the beginning of an effort to expand the analysis to include individuals of the same age who do not have photosensitivity.

**Methods:**

A total of 26 facial portraits of TTD paediatric patients with Non‐photosensitivity from the literature were analysed using computer‐aided technologies, and their facial features were examined through a detailed clinical review.

**Results:**

Distinct facial features were identified in both Photosensitive and Non‐photosensitive TTDs.

**Conclusion:**

The present study has comprehensively elucidated the facial features in TTDs, encompassing the Non‐photosensitive clinical spectrum.

## Graphical abstract

This is a novel study regarding the facial features of paediatric patients with the Non‐photosensitive TTD clinical spectrum.

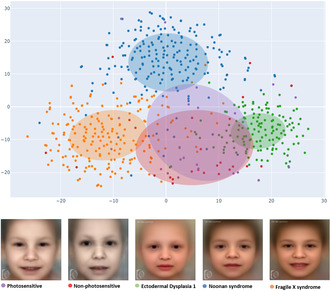

## 1 INTRODUCTION

Non‐photosensitivity defines a wide clinical and genetic spectrum among trichothiodystrophies (TTDs). This is a rare subset of genodermatoses primarily characterized by brittle, sulphur‐deficient hair and a ‘tiger tail banding’ hair appearance under polarizing microscopy.

Using novel molecular technologies, several causative genes have been identified, which have often resolved cases of previously reported patients and highlighted genetic heterogeneity. Some of them are involved in cellular cycle regulations as well as in transcription, RNA processing, and protein synthesis. The 3 forms with photosensitivity now include additional TTDs, ranging from the first ones described, such as Amish brittle hair brain and Sabinas brittle hair syndrome (type 4, TTD4, MIM#234050; *MPLKIP/TTDN1*, MIM*609188), to the more recently identified types: 5 (TTD5, MIM#300953; *RNF113A*, MIM*300951), 6 (TTD6, MIM#616943; *GTF2E2* MIM*189964), 7 (TTD7, MIM#618546; *TARS1*, MIM*187790), 8 (TTD8, MIM#619691; *AARS1*, MIM*601065) and 9 (TTD9, MIM#619692; *MARS1*, MIM*156560). The recently identified Microcephaly, Developmental Delay, Brittle Hair Syndrome (MDBH, MIM#618891), caused by variants in *CARS1* (MIM*123859), has also been considered.

Following our previous study on Photosensitive phenotype (Pascolini et al., 2023), we analysed the facial characteristics of TTD patients with Non‐photosensitivity during childhood to enhance and broaden our understanding of this unique topic.

For this purpose, we have selected all published paediatric patients within this clinical spectrum, detailing the facial features and analysing frontal portraits available with computer‐aided technologies.

This consecutive research is the first on patients with Non‐photosensitive TTDs, illustrating interesting results and supporting our earlier evidence.

## 2 SUBJECTS AND METHODS

We researched indexed articles in the online database PubMed (https://pubmed.ncbi.nlm.nih.gov), focusing on patients aged 1–14 years, without any specific ethnicity preference, and with accessible frontal facial photographs. We used the following terms for the research: trichothiodystrophy, trichothiodystrophy 4, 5, 6, 7, 8, 9. Also, key words, such as BIDS, IBIDS, SIBIDS, Sabinas brittle hair, Pollitt and Amish Brittle Hair Brain syndromes (ABHBS), microcephaly developmental delay, and brittle hair syndrome (MDBH) were used for patient selection and identification of causative genes. From this initial phase, we identified a total of 34 patients. Of these, we excluded 2 patients because their age at the moment of the photo acquisition was not specified; 2 patients were excluded because the image could not be analysed using the CLINIC application of the Face2Gene platform (F2G, www.face2gene.com) (vs21.5.0), powered by FDNA Inc., (Boston, USA), which we examined and represent a part of this study; 2 were excluded because there was no available information about their facial features, and their published portrait was not suitable for F2G; 2 of them were excluded because the TTD type (Photosensitive vs. Non‐photosensitive) was not clear; and one more because the individual was 15 years old.

Then, we reached out to the final number of 26 individuals, whose facial details were carefully recorded (Table 1) (Alfandari et al., 1993; Arbisser et al., 1976; Bracun et al., 1997; Civitelli et al., 1989; Corbett et al., 2015; Gillespie et al., 1988; Heller et al., 2015; Hersh et al., 1993; Hordinsky et al., 1987; Howell et al., 1981; Jorizzo et al., 1982; Kuschal et al., 2016; La Serna‐Infantes et al., 2018; Liang et al., 2005, 2006; Lynch et al., 1995; Mendelsohn et al., 2020; Nakabayashi et al., 2005; Pande et al., 2020; Price et al., 1980; Przedborski et al., 1990; Rizzo et al., 1992).

**TABLE 1:** Facial features of Non‐photosensitive TTD patients in childhood.

TTD gene	No.	Reference	Individual	Clinical classification/variant	Age	Gender	Skull	Eye	Eyebrows	Eyelashes	Lips/mouth/palate	Ear	Nose	Chin/cheeks	Teeth	F2G ID
na	1 2	Arbisser et al. (1976); Howell et al. (1981)	Case 1	Sabinas brittle hair syndrome	6 years	F	na	+ (epicanthal folds) [epicanthus]	+ (marked reduction) [sparse]	+ (marked reduction) [sparse]	na	na	na	na	na	972307
			Case 2	Sabinas brittle hair syndrome	5.5 years	M	na	na	+ (marked reduced) [sparse]	+ (marked reduced) [sparse]	na	na	na	na	+ (crowded) [dental crowding]	972701
	3 4	Price et al. (1980)	Case 1	TTD	8.5 years	M	+ (elongated head) [turricephaly]	+ (prominent bilateral epicanthal folds) [epicanthus]	+ (broken stubble and rarely visible) [sparse]	+ (lower: sparse and broken) [sparse]	na	+ (protruding)	na	+ (receding chin) [retrognathia]	‐	970036
			Case 2	TTD	5 years	M	na	+ (prominent eyes) [proptosis]	+ (scanty) [sparse]	+ (scanty) [sparse]	na	+ (protruding)	+ (beaked) [convex nasal ridge]	+ (receding chin) [retrognathia]	+ (many caries, yellowish discolouration)	970037
	5	Jorizzo et al. (1982)	1	BIDS	8 years	M	na	na	+ (sparse)	+ (sparse)	na	+ (bunched up) [overfolded helix]	na	na	‐	972310
	6	Hordinsky et al. (1987)	1	BIDS	22 m	M	na	na	na	na	na	na	na	na	na	770584
	7	Gillespie et al. (1988)	Case 1	TTD	6 years	F	na	+ (small alternating convergent squint)	+ (short, sparse and disordered) [sparse]	+ (very few intact) [sparse]	na	+ (protruding)	na	+ (receding chin, plump cheeks) [retrognathia, full cheeks]	‐	770578
	8	Civitelli et al. (1989)	1	IBIDS	9.8 years	M	+ (dolichocephaly)	na	na	‐	na	+ (protruding and low‐set)	na	na	‐	770574
	9	Alfandari et al. (1993)	1	TTD	5 years	M	na	na	+ (short, sparse) [sparse]	+ (short, sparse)	na	na	na	na	‐	972308
	10	Hersh et al. (1993)	1	SIBIDS	20 m	M	+ (microcephaly, triangular face, prominent head biparietally) [prominent forehead]	na	+ (short, sparse) [sparse]	+ (short, sparse)	na	na	na	na	‐	770583
	11	Lynch et al. (1995)	1	TTD	3 years	M	+ (head circumference <3rd centile)	na	+ (sparse)	+ (sparse)	na	na	na	na	+ (severe dental caries, single fused lower central incisor) [single maxillary central incisor]	770587
	12	Bracun et al. (1997)	One sibling	TTD	7 years	F	+ (dysmorphic face)	na	+ (sparse)	+ (sparse)	na	+ (protruding)	na	na	na	973168
	13	Liang et al. (2005)	1	TTD	3 years	F	na	na	na	na	na	na	na	na	na	969296
	14	Liang et al. (2006)	TTD353BE	TTD	14 m	F	na	na	na	na	na	na	na	na	na	979297
TTD4 MPLKIP (TTDN1) (MIM*234050)	15	Heller et al. (2015)	TTD402BE	c.2T>G; c.2T>C	14 years	M	na	na	+ (sparse lateral)	na	+ (high arched palate) [high palate]	na	na	+ (retrognathia)	na	770579
	16		TTD480BE	deletion of ~120 kb; c.277delG	4 years	F	na	+ (strabismus, epicanthal folds, mild telecanthus) [epicanthus]	+ (sparse)	+ (broken) [sparse]	na	na	+ (high nasal bridge) [prominent nasal bridge]	+ (micrognathia)	na	770580
	17		TTD487BE	c.277delT; deletion of ~92 kb	2 years	F	na	+ (epicanthal folds, mild telecanthus) [epicanthus]	+ (sparse)	+ (sparse)	na	+ (simplified) [stahl]	na	+ (micrognathia)	na	770581
	18		TTD488BE	c.277del; deletion of ~92 kb	1 year	M	na	+ (epicanthal folds) [epicanthus]	+ (sparse)	+ (sparse)	na	+ (left crease, simplified) [stahl]	na	na	na	770582
	19	Nakabayashi et al. (2005);Przedborski et al. (1990)	Moroccan sibling; Patient 3 (I.E.)	c.137_138delGG; c.137_138delGG	13 m	F	+ (microcephaly)	+ (hypotelorism, epicanthal folds) [closely spaced eyes, epicanthus]	+ (absent)	+ (absent)	+ (high arched palate) [high palate]	+ (poorly rimmed ears) [stahl]	na	+ (micrognathia)	‐	965621

	20	Nakabayashi et al. (2005);Rizzo et al. (1992)	Patient 6474; Patient 1 (V.V.)	deletion of part of exon 1 and the entire exon 2 (homozygous)	3 years	F	na	+ (horizontal palpebral fissures, epicanthal folds) [epicanthus]	+ (sparse)	+ (sparse)	na	+ (large)	+ (short with large, depressed nasal root, thick alae) [depressed nasal bridge, thick ala nasi]	+ (mild retrognathia)	+ (hypoplastic, especially on the lower arch)	770589
	21	Shah et al. (2016)	IV 4	c.339+1G>A; c.339+1G>A	8 years	M	na	na	+ (sparse)	+ (sparse)	+ (high arched palate) [high palate]	na	na	na	+ (irregular, hypodontia)	770590
	22	La Serna‐Infantes et al. (2018)	1	arr[hg19]7p14.1 (40,140,770‐40,265,451)x0 (homozygous)	8 years	F	+ (microcephaly, asymmetric face)	na	+ (sparse)	+ (sparse)	+ (hypoplastic supraciliary/zygomatic arches, downslanting mouth corners) [cheekbone underdevelopment, mouth downturned corners]	na	+ (anteverted nares and thick nasal root) [wide nasal bridge]	na	+ (dental caries and multiple teeth decay)	965308
23	Pande et al. (2020)	Proband 1	c.229del; c.229del	13 years	M	+ (mild facial asymmetry)	+ (telecanthus)	+ (sparse with absent lateral half)	+ (sparse with absent lateral half)	+ (thick vermilion border of lower lip, long philtrum) [lower lip thick vermilion]	na	+ (depressed nasal bridge)	na	+ (small and mal‐aligned teeth) [microdontia]	771411
TTD5 *RNF113A* (MIM*300951)	24	Corbett et al. (2015)	Patient 1 (IV‐5)	c.901C>T (hemizygous)	8 years	M	+ (microcephaly, high forehead) [high anterior hairline]	na	+ (sparse, brittle, slow growing, absent outer) [sparse]	+ (sparse, brittle, slow growing)	+ (broad mouth, macrostomia) [wide mouth]	+ (extra auricular crus)	na	+ (prominent chin) [pointed chin]	+ (widely spaced primary teeth)	770576
	25	Mendelsohn et al. (2020)	Patient 2	c.897_898delTG (hemizygous)	2 years	M	+ (microcephaly, a triangular face with a broad forehead)	na	+ (absent)	+ (absent)	+ (wide mouth)	+ (pointed and cupped, posteriorly rotated, and low‐set) [satyr, increased posterior angulation, cupped and low‐set]	na	na	na	965310
TTD6 *GTF2E2* (MIM*189964)	26	Kuschal et al. (2016)	TTD379BE	c.448G>C; c.448G>C	10 years	M	+ (microcephaly)	na	na	na	na	+ (low‐set)	na	+ (mild micrognathia)	na	965306
Total: 26						16/26 M; 10/26 F	11	10	21	20/21	7	13	5	10	15	

*Note*: The terms are reported as in the original descriptions. Some terms have been indicated according to the American Journal of Medical Genetics (AJMG) standard terminology (Elements of Morphology, 2009) (in square brackets).

Abbreviations: BIDS, brittle hair, intellectual impairment, decreased fertility, short stature; F, female; F2G ID, Face2Gene identity; IBIDS, ichthyosis, brittle hair and nails, intellectual impairment, short stature; M, male; m, months; na, not available; SIBIDS, osteosclerosis, ichthyosis, brittle hair, impaired intelligence, decreased fertility, short stature.

The images were then processed using the research application of F2G. With this tool, we performed a binary comparison analysis involving two experiments. We compared 26 images with those of 26 unaffected individuals matched for age and ethnicity, as well as with 22 Photosensitive patients from our previous study (Pascolini et al., 2023).

**FIGURE 1:**
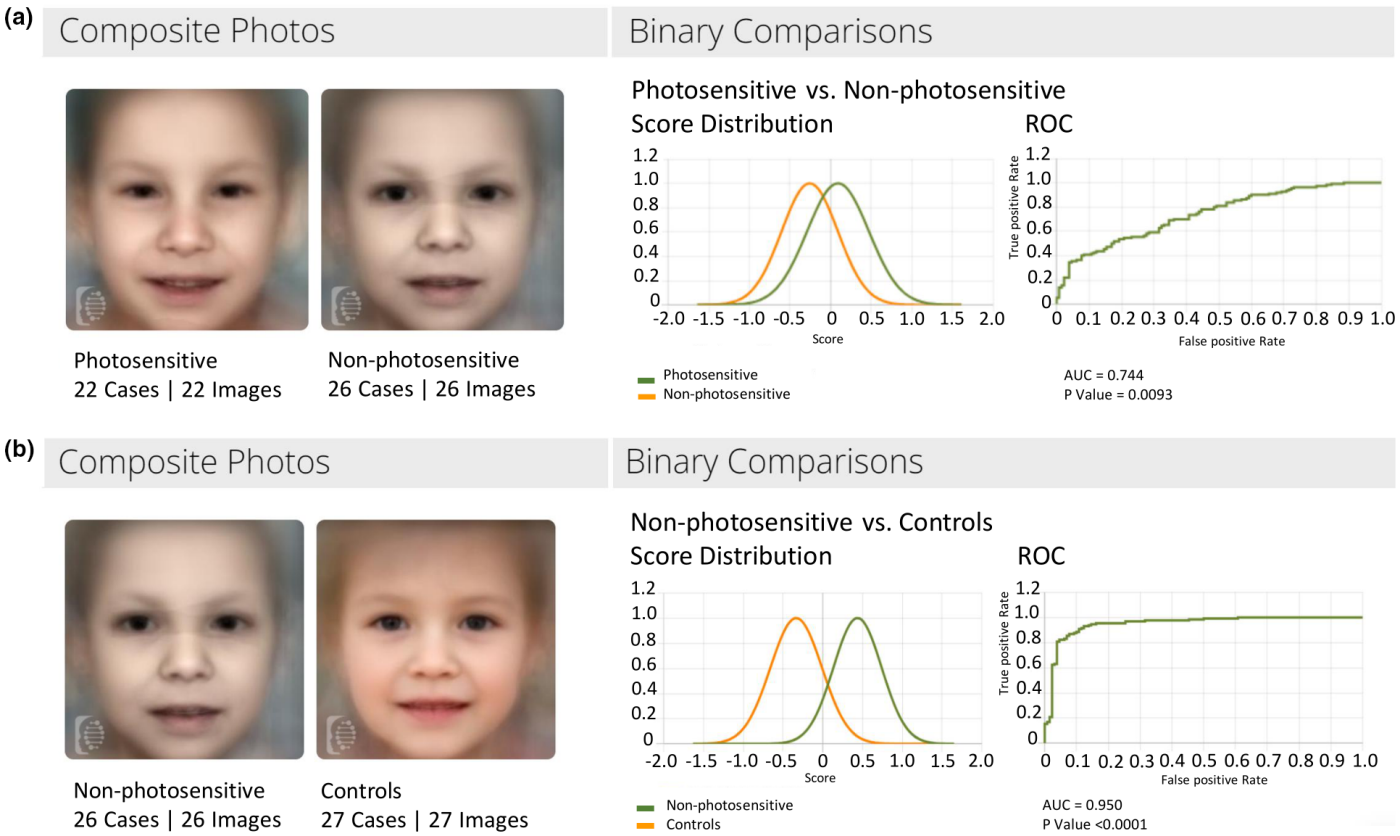
DeepGestalt analysis results. (a) Non‐photosensitive versus Photosensitive and (b) Non‐photosensitive versus Controls experiments. Score distribution and ROC curve, showing the comparison results, are provided (AUC = 0.744, *p*‐value 0.093 and AUC = 0.950, *p*‐value < 0.0001, respectively).

In the 2 DeepGestalt analyses (Non‐photosensitive vs. Photosensitive; Non‐photosensitive vs. Controls), the quality of separation was evaluated by creating composite images of both groups and measuring the area under the curve (AUC) of the receiver operating characteristic (ROC) curve (Figure 1a,b).

By applying the GestaltMatcher algorithm, we encoded a patient photo into a 320‐dimensional vector, called facial phenotype descriptor (FPD), which can be interpreted as a coordinate in the clinical face phenotype space (CFPS). Distances between different FPDs in the CFPS define syndromic similarity—smaller distances indicate higher similarity.

By using GestaltMatcher, it is possible to classify syndromes, delineate new phenotypes, and cluster patients with similar clinical presentations. In the resulting pairwise comparison matrix (PCM), we compared the images of the target cohort cases (*x* and *y* axes) with the GestaltMatcher gallery (4300 images), which resulted in clustered similarity ranks.

Using a dimensionality reduction method to convert the 320‐dimensional vectors to2 dimensions, allowed us to visualize them in a 2D scatterplot using t‐SNE visualization.

Each data point corresponds to a patient photo. This visualization includes the target cohort and the top 3 most similar rare syndromes, each with at least 7 patient photos available in the GestaltMatcher database. The confidence ellipses have a radius of one standard deviation from each cluster's centroid.

The closer these data points are, the more similar their facial dysmorphology is.

## 3 RESULTS

The present study cohort consists of 16 males and 10 females. The facial details shown in Table 1 will be used to describe the total number of individuals with such feature in this paragraph. Clinical terms have been listed in the table as originally reported. However, we added in square brackets the actually used craniofacial standard terminology (Allanson et al., 2009; Carey et al., 2009; Hall et al., 2009; Hennekam et al., 2009; Hunter et al., 2009). Thus, skull morphology was anomalous in 11 individuals, with 11 displaying various characteristics, including microcephaly (7), triangular face (2), dolichocephaly, prominent biparietal head, high forehead, broad forehead, and asymmetric face (each in 1).

Eye anomalies were diagnosed in 10 individuals: epicanthal folds in 7 (prominent 1), telecanthus in 3; strabismus, hypotelorism, prominent eyes, and small alternating convergent squint in 1 (each).

Eyebrows were abnormal in 21 cases: they appeared sparse (9), short and sparse (3), marked reduced (2), absent (2) and in 1 (each) broken stubble and rarely visible; short, sparse and disordered; sparse lateral; sparse with absent lateral half; sparse, brittle, slow growing and absent outer. Eyelashes were involved in 20/21 and were sparse (8), marked reduced (2), short and sparse (2), absent (2), sparse and broken; very few intact; broken, sparse with absent lateral half; sparse, brittle and slow growing, each present in 1. Abnormal eyebrows and/or eyelashes were observed in all patients except one.

The oral region was involved in 7 cases: a high‐arched palate was identifiable in 3, analogously to a wide mouth (including broad mouth and macrostomia); one individual had hypoplastic supraciliary/zygomatic arches and down‐slanting mouth corners; a thick vermilion border of the lower lip and a long philtrum were observed in each case. Thirteen (13) patients exhibited ear anomalies: they were protruding in 4, simplified in 2, low‐set, protruding with low‐set, large in 1 (each). Nose malformations were ascertained in 5: abnormal nasal bridge (depressed, high) was registered in 2; beaked nose, short nose with large and depressed nasal root, thick alae, anteverted nares and thick nasal root were identifiable in 1 (each).

Ten (10) patients had chin and cheek anomalies. Micrognathia was present in 4, receding chin in 3, retrognathia in 2, plump cheeks with prominent chin in 1 (each).

For 15 patients, information about their teeth was available. Eight out of the fifteen had impairment: they were crowded, small, and misaligned, with widely spaced, hypoplastic teeth, especially in the lower arch in 1 (each); in the same proportion, there was yellowish discolouration, a single fused lower central incisor, hypodontia, and multiple decays. Caries was diagnosed in 3 teeth.

The Non‐photosensitive versus Photosensitive analysis showed a *p*‐value of 0.093, whereas in the Non‐photosensitive TTD versus Controls experiment, this parameter resulted in <0.0001 (Figure 1a,b).

In the PCM of the GestaltMatcher analysis, the number in each box represents the facial phenotype similarity rank achieved (Figure S1). Dark green values (indicating a low rank) in this matrix indicate higher similarity in facial phenotypic features. For example, if the case IDs are the same on the *x* and *y* axes, the value is 0. This value represents the similarity rank achieved when each photo is compared to itself. In addition, a clustering method was applied to the matrix to group similar ranks together. If we look at the PMC, and we take the top level that splits the cohort in two, we see that the majority of the case IDs on the right belong to the Non‐photosensitive TTD group, whereas the majority of the case IDs on the left belong to the Photosensitive TTD group, but there is still some overlap (Figure S1).

**FIGURE 2:**
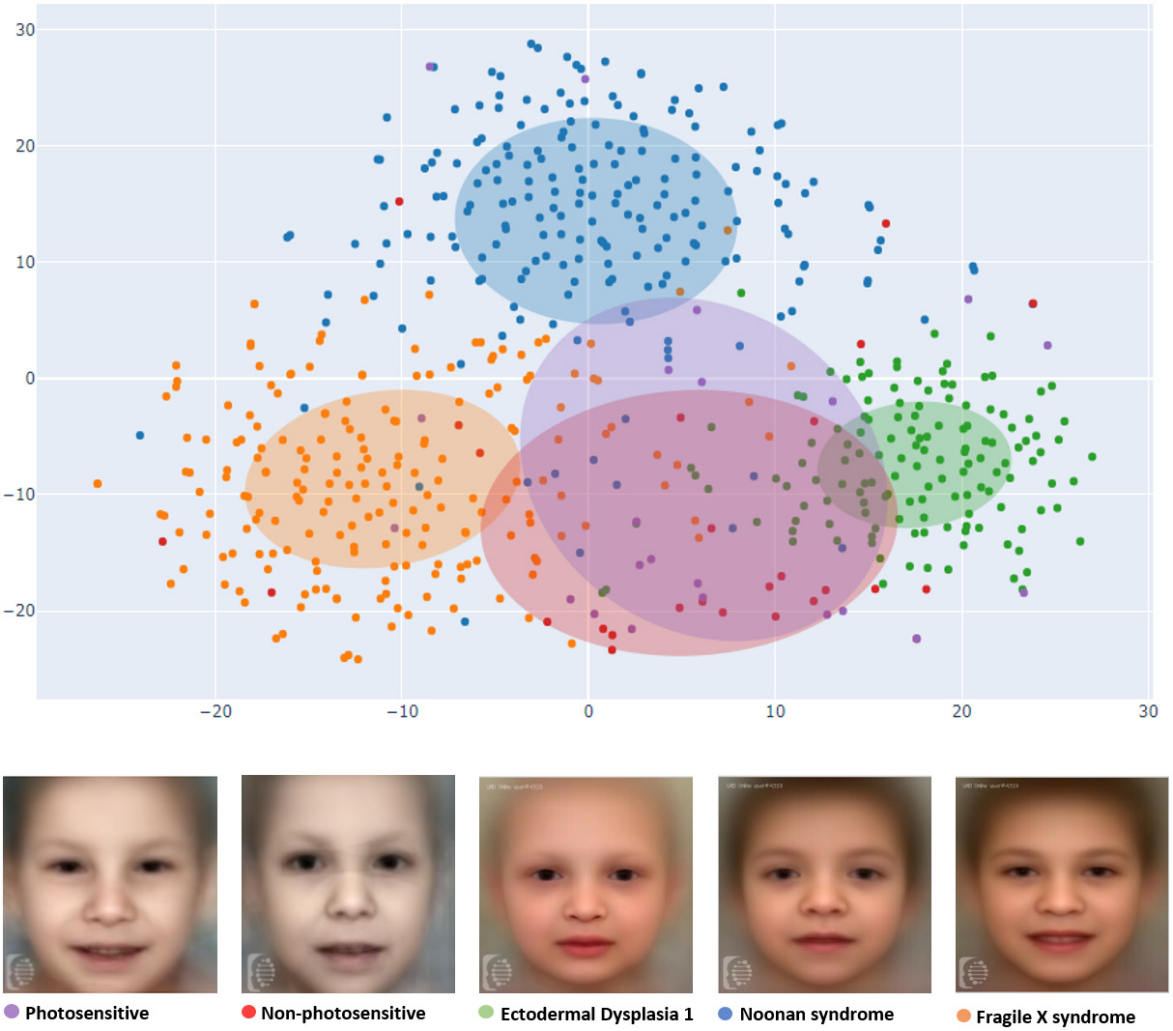
Results of the GestaltMatcher experiment. t‐SNE visualization showing Photosensitive (in violet), Non‐photosensitive (in red) TTDs and the most similar syndromes when compared using the GestaltMatcher database: Ectodermal Dysplasia 1 (in green), Noonan (in blue) and Fragile X (in orange) syndromes.

In the t‐SNE representation, Figure 2 shows that both subgroups clusters are separate from the top 3 most similar syndromes in the GestaltMatcher database: Noonan (NS), Fragile X (FXS) and Ectodermal Dysplasia 1 (Hypohidrotic, X‐linked; XHED) syndromes. They do not seem to separate among themselves, indicating they are likely quite similar in terms of facial phenotype (Figure 2).

## 4 DISCUSSION

This represents a follow‐up study to one we recently published on the facial phenotype of paediatric patients with Photosensitive TTDs. In this second research, we have considered the Non‐photosensitive clinical spectrum, a wide genetically determined group of TTDs. In recent years, the introduction of novel molecular techniques has enabled us to identify the underlying cause of previously described patients and additional individuals, as well as discover new genetic determinants. Based on the molecular defect, four Non‐photosensitive TTDs can be identified: the genes involved have diverse functions and drive distinct molecular pathways, highlighting the strong genetic heterogeneity of this group of conditions. *MPLKIP*, which causes TTD4, is the most prevalent gene in this cohort (10/26). It encodes an M‐phase specific PLK1‐interacting protein, involved in the regulation of mitosis and cytokinesis (Zhang et al., 2007), as well as in RNA processing (Theil et al., 2023). The other two proteins, *RNF113A* (Ring Finger Protein 113A) and *GTF2E2* (General Transcription Factor IIE Subunit 2), are less common and are respectively associated with TTD5 and TTD6. The first point of interest is the unique TTD gene located on chromosome X, which is a component of the spliceosome (Haselbach et al., 2018; Zhang et al., 2018) and plays a crucial role in the DNA repair process (Brickner et al., 2017). Meanwhile, *GTF2E2* is crucial for promoter clearance by RNA polymerase and transcription activation (Peterson et al., 1991). However, the present study did not include patients with variants in the recently identified genes *AARS1* (Alanyl‐tRNA synthetase 1) and *MARS1* (Methionyl‐tRNA synthetase 1), causing TTD8 and TTD9 (Botta et al., 2021). This is because in the first case, we did not find any paper with images of children with *MARS1* mutations (to the best of our knowledge), whereas in the only paper reporting a paediatric individual with an *AARS1* variant, the age of the patient in the facial portrait was not clear (Botta et al., 2021). We also did not include the patient mentioned in Jackson et al. (1974) for the same reason, even though he represented one of the first clinical descriptions of a TTD4 patient in the Amish population, who identified the ABHS. In addition, information about his facial characteristics was not provided, and the image was not analysable with the CLINIC application of F2G. This also occurred with the two patients studied by King et al. (1984), who were diagnosed with TTD4 but were also noted to have photosensitivity, creating a source of confusion. This supported their exclusion from our earlier study. Moreover, their portraits were not suitable for the DeepGestalt analysis. Finally, we did not find facial photos of paediatric patients with variants in *TARS1* (Threonyl‐tRNA synthetase 1) and *CARS1* (Cysteinyl‐tRNA synthetase 1), associated respectively with TTD7 (Theil et al., 2019) and MDBH (Kuo et al., 2019).

The facial phenotype of the Non‐photosensitive clinical spectrum in childhood has been analysed in the present study. This involved compiling the clinical details of previously published patients (from 1976 to 2020) and conducting additional experiments using the novel DeepGestalt and GestaltMatcher technologies.

The data we have obtained (Table 1) are consistent with our previous study, identifying skull anomalies, eye problems, oral defects, abnormal ear, nose, chin, and cheeks as the most prevalent facial features (100%). This is followed by eyebrows and eyelashes (95%) and teeth involvement (53%). This can lead to the recognition of a distinctive facial phenotype in TTD patients, suggesting a recurring pattern of facial abnormalities. A careful observation of these clinical features would be desirable in the diagnostic process.

This was also supported by the binary comparison analysis, which identified a different facial morphology between Non‐photosensitive TTDs and controls, highlighting the good performance of the F2G platform in differentiating the two facial profiles. An overlap between the two main types of TTDs in the second experiment (Non‐photosensitive vs. Photosensitive) is demonstrated by the *p*‐value, which was not significant, indicating that both groups are not separable and then that their facial phenotype is similar (Figure 1a,b).

Notably, the GestaltMatcher algorithm revealed two similar syndromes that were not identified in the previous study (Figure 2), including XHED and NS. Except for the first disorder, which can be easily understood as a similar condition due to being a genodermatosis with impairment of ectodermal derivatives, it would be interesting to further explore the similarities with the other disorder, which is not classically considered a genetic disease primarily affecting the skin. Potential clinical intersection points can be represented by some dermatological signs identifiable in NS (woolly‐like hair, low posterior hairline, ulerythema ophryogenes). If this is associated with a possible interaction between the underlying molecular pathways, it would be clarified.

Interestingly, XHED overlaps with both TTDs, whereas FXS and NS respectively with the Non‐photosensitive and Photosensitive type (Figure 2). This could be considered for a correct differential diagnosis.

In conclusion, we characterized the facial phenotype of children within the Non‐photosensitive TTD spectrum, building upon and expanding our previous study.

TTDs in their two main clinical forms, are characterized by a distinctive facial appearance in paediatric patients.

## AUTHOR CONTRIBUTIONS

GP conceived the study, performed the literature review with clinical images selection, wrote the manuscript, performed the DeepGestalt experiment, coordinated the GestaltMatcher analysis, give the final approval of the manuscript; ML, FG, LF contributed to perform the literature review; GD contributed to write the manuscript and give the final approval; BD give the final approval of the manuscript.

## CONFLICT OF INTEREST STATEMENT

The authors declare that there is no conflict of interest regarding this study.

## FUNDING INFORMATION

This work did not receive any funds.

## ETHICS STATEMENT

This study was performed according to the Helsinki Declaration. Approval from an internal review board was not necessary since the study is based on a literature review.

## Supporting information


Figure S1.

## Data Availability

Data regarding this study are available from the corresponding author upon reasonable request.
