# Supplementary figures and images for: The face of Non‐photosensitive trichothiodystrophy phenotypic spectrum: A subsequent study on paediatric population
Source: Mol Genet Genomic Med. 2024 Aug 9;12(8):e2501. doi: 10.1002/mgg3.2501 (PMC11310551; doi:10.1002/mgg3.2501)

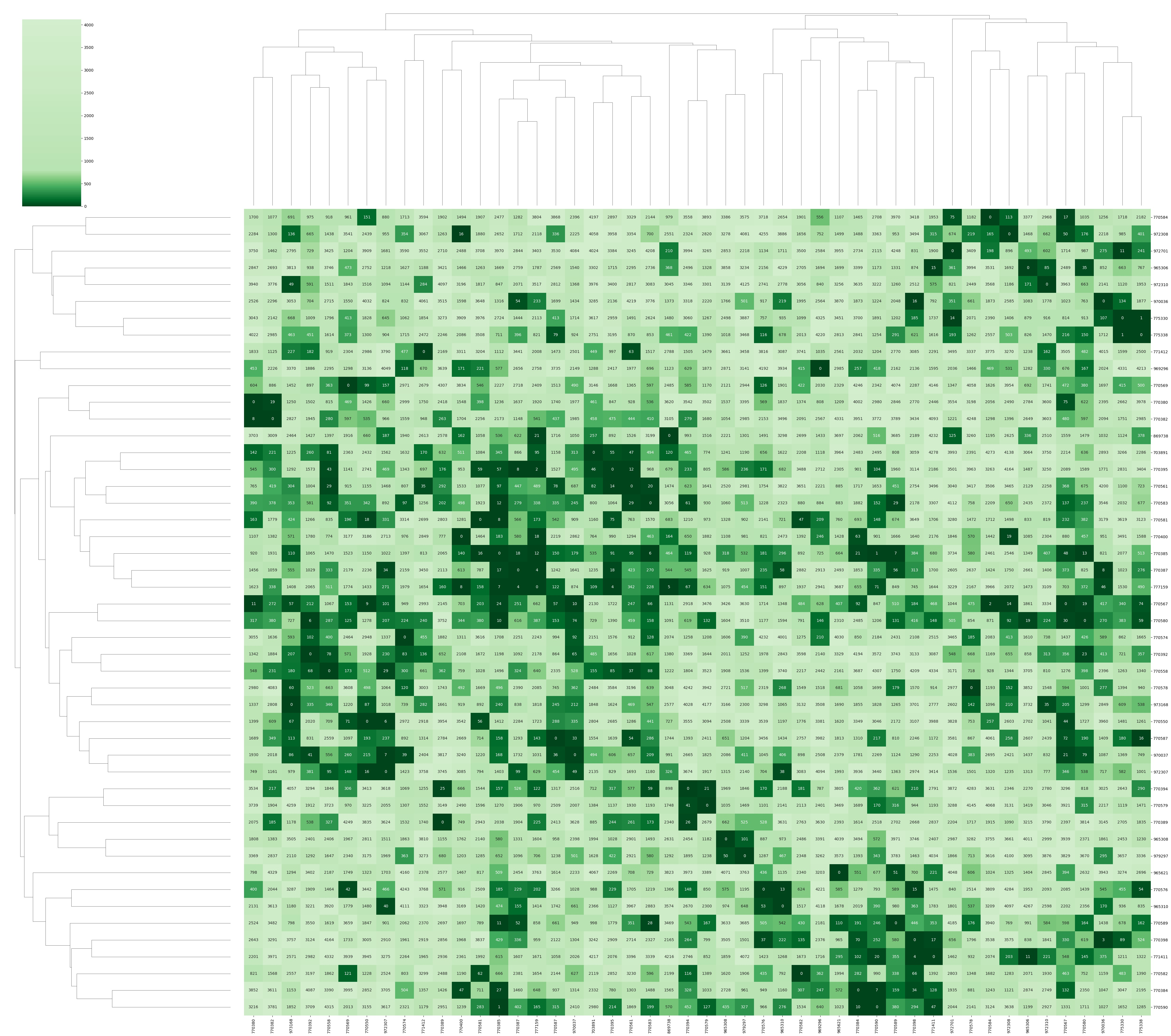

Supplement: Supplementary file 1 — Figure S1. [file MGG3-12-e2501-s001.tif]
